# A bibliometric and subject analysis of 3300 most‐cited articles in dentistry

**DOI:** 10.1002/cre2.633

**Published:** 2022-08-07

**Authors:** Ghazaleh Daryakenari, Zahra Batooli

**Affiliations:** ^1^ Department of Restorative dentistry Kashan University of Medical Sciences Kashan Iran; ^2^ Social Determinants of Health (SDH) Research Center, Faculty of Health Kashan University of Medical Sciences Kashan Iran

**Keywords:** bibliometrics, dental research, dentistry, oral medicine

## Abstract

**Objectives:**

This study aims to analyze the publications that have studied top‐cited articles in dentistry.

**Material and Methods:**

The study is applied research in terms of the purpose and scientometrics descriptive in terms of type, which has been done using the Retrospective Bibliometric Analysis. To retrieve the 100 most‐cited studies in Scopus, an advance search was used. The search results indicate that 33 articles focused on analyzing the 100 most‐cited publications on oral and dental subjects. The bibliographic information, including author, journal, country, institution, citation count, and citation density was analyzed. Then the full text of the articles was reviewed to identify the most productive journal, country, and institute in publishing the 100 most‐cited articles and also article type, study design, level of evidence, and the most important subjects among the 100 most‐cited papers.

**Results:**

In these 33 articles, the topics of 3300 most‐cited articles were introduced, as well as the introduction of the core journals, countries, and institutes in publishing top‐cited articles. The most common research topics in the field of dentistry cover a range of dental public health and pediatric dentistry to adhesive restorative dentistry and implantology. Dental materials, oral medicine, and pathology seem like inseparable topics of common research in dentistry. *J Dent Res*, which was mentioned in nine articles, was introduced as the journal with the publication of articles of highest citation. *J Clin Periodontol* and *Oral Surgery*, *Oral Medicine*, *Oral Pathology*, and *Oral Radiology* were in the second place, being mentioned in five articles. The USA, and two institutions *the University of Texas*, and *the University of Michigan* are orderly core country and institute for the production of most‐cited articles.

**Conclusion:**

Researchers and specialists can get familiar with the most productive author, journals, countries, and different institutes for publishing high‐quality articles in the field of oral and dental subjects by the means of the results of this article. Furthermore, the results of this study ranked the most cited article topics, which are of interest for publication, demonstrating the future map road oral and dental research.

## 1 INTRODUCTION

The quantitative and qualitative analysis of scientific findings resulting from research activities will enable the officials and managers to take advantage of the manpower and financial resources with the lowest budget, using that data, to optimize the socioeconomic structure of the country. Thus, the financial and bureaucratic programs of the research system require evaluation of the scientific outputs by the means of scientometric methods (Aminpour et al., 2008).

Furthermore, analysis of science products is a suitable tool for better policy making and planning, understanding the past situation, determining the research priorities, and identifying the weaknesses and deficiencies in the production of scientific information (Bazrafshan & Mostafavi, 2011). One of the efficient ways of analyzing the scientific output and the overall research status is the evaluation of the articles indexed in databases. An analysis of this kind can somewhat determine the amount of research carried out by every country, organization, scientific field, and each person, as well as the pertaining process.

On the other hand, citation analysis is a very important indicator in scientometric research. The citation number will affect the impact factor of the journal of publication; this has also been representative of the article's confirmation, efficiency, quality, or the article's and authors' reputation (Joyce et al., 2014; Moed, 2009; Paladugu et al., 2002; Shuaib et al., 2015). The articles with higher citations are a turning point in any field and can affect the research and clinical approach. The 100 most‐cited articles in any field are considered the most effective ones in that subject. Scientometric analysis of the most affecting publications in any field will enhance our knowledge of the research progression and subjects of interest in that area. This type of analysis, which has received special attention in recent years, shows the publication procedure, science evolution, and evidence‐based operation of a subject throughout the years. The collection of most‐cited publications might help the physicians and specialists of any field, to have a better realization of the nature of that subject. The citation count might not be a criterion for quality assessment. However, it is utilized to evaluate the influence of such an article on the scientific society so that the articles with higher quality will have a substantial range of citations compared to articles of lower quality (Bornmann & Daniel, 2008). Keeping in mind the importance of such matters in recent years, different research is carried out about the analysis of the 100 most‐cited publications in diverse fields, which further enlightens the importance of considering these kinds of research, including analysis of the top 100 articles on vestibular schwannoma (Martinez‐Perez et al., 2021), cariology (Baldiotti et al., 2021), nursing student education (Chang et al., 2021), cerebral vasospasm (Elarjani, Almutairi, Alhussinan, Alzhrani, et al., 2021), osteoporosis (Gao et al., 2020), ophthalmology (Koh et al., 2020), craniosynostosis (Elarjani, Almutairi, Alhussinan, Alturkistani, et al., 2021), bladder cancer (Mainwaring et al., 2020), imaging of the spine (Das et al., 2019), immunotherapy for childhood leukemia (Zhong et al., 2019), pediatric traumatic brain injury (Karydakis et al., 2020), and urogynecology (Gupta et al., 2020).

The field of dentistry and related sciences are in direct relation to human wellbeing, thus making this field very important. Of the high costs of education and research in the field, it is only fair to plan properly. Evaluating different aspects of the research in this area can give a direction to future research, help planning for further development in different dental sciences, optimal budget and facility allocation, and last but not least, upgrade the quantity and quality of the production in this field. Keeping that in mind, some publications have focused on analyzing the 100 most‐cited articles in the field of dentistry and related sciences. The analysis of these articles will allow the specialists to be acquainted with the core authors, countries, institutes, and journals, as well as the most interesting and effective subjects in this field. Thus, this study aims to analyze the publications that have studied the most‐cited articles on dentistry and related sciences. A bibliometric and subject analysis of the most‐cited articles on this area can gather valuable information for the future roadmap of dental research.

## 2 MATERIALS AND METHODS

This was a scientometrics descriptive study, which has been done using the retrospective bibliometric analysis. Bibliometric analysis is one way of scientific study, which aims to identify the Performance Indicators of research based on the quantitative analysis of the scientific product (Moed, 2009). To retrieve the 100 most‐cited studies in the Scopus database, the advanced search sector was used. Scopus is currently the greatest universal database that covers a comprehensive amount of subjects concerning medical, biology, and social sciences, thus this database was chosen. In the TITLE‐ABS field, the (100 OR hundred) and (cited OR citation) keywords are combined with the w/5 proximity operator. This study, which was performed on January 24, 2021, retrieved 883 articles. To retrieve articles that have analyzed the 100 most‐cited articles in the dental field, the result of the search was limited to dentistry in the subject area section. Finally, it was evident that 33 articles have focused on the analysis of the 100 most‐cited papers in the dental field. Similar to other studies, the eligibility of articles is based more on accuracy and relevance to the purpose of the study than on the quality determined (Ball et al., 2011; Pawson et al., 2004; van Hooft et al., 2017).

Then, the full text of these 33 articles was retrieved and used to derive the following:

- The database used to retrieve articles;
- The most productive journals, countries, and institutions in publishing 100 most‐cited papers;
- Article type, study design, and level of evidence among 100 most‐cited papers;
- And the most important subjects.

## 3 RESULTS

The search results indicate that 33 articles focused on analyzing the 100 most‐cited publications on oral and dental subjects. The process of publication was ascending from 2011 to 2021. Half of the articles were published in 2019 and 2020. The year 2020 with 12 articles has the largest number. According to the author, there were 138 authors who participated in publishing these articles. *Ahmad, Paras* from *AO Research Institute Davos, Switzerland* is the most productive author with four articles. In second place are 10 authors with three articles each. These authors are affiliated with 81 universities and institutes. According to the institution, nine institutions each with three articles are the most productive institution. The findings showed that 33 articles were published in 27 journals, all in the field of dentistry. These 33 articles were published with the cooperation of 20 countries. Saudi Arabia is in first place with 8 articles. Brazil and Malaysia are to follow with five articles each. The 33 publications have been cited 425 times. The mean citation count of the nonzero incidents was 13.7. The most‐cited articles based on citation count and citation density are presented in Table 1.

**Table 1:** Top articles based on number of citation and citation density

No. of citation							
**Rank (Citation)**	**Author**	**Title**	**Year**	**Journal**	**Citation**	**Citation density**	**Rank (CY)**
1	Fardi A., Kodonas K., Gogos C., Economides N.	Top‐cited articles in endodontic journals	2011	Journal of Endodontics	75	7.50	4
2	Feijoo J.F., Limeres J., Fernández‐Varela M., Ramos I., Diz P.	The 100 most‐cited articles in dentistry	2014	Clinical Oral Investigations	67	9.57	1
3	Jafarzadeh H., Sarraf Shirazi A., Andersson L.	The most‐cited articles in dental, oral, and maxillofacial traumatology during 64 years	2015	Dental Traumatology	31	5.17	7
4	Pena‐Cristóbal M., Diniz‐Freitas M., Monteiro L., Diz Dios P., Warnakulasuriya S.	The 100 most‐cited articles on oral cancer	2018	Journal of Oral Pathology and Medicine	26	8.67	2
5	Hui J., Han Z., Geng G., Yan W., Shao P.	The 100 top‐cited articles in orthodontics from 1975 to 2011	2013	Angle Orthodontist	24	3.00	8
6	Tarazona B., Lucas‐Dominguez R., Paredes‐Gallardo V., Alonso‐Arroyo A., Vidal‐Infer A.	The 100 most‐cited articles in orthodontics: A bibliometric study	2018	Angle Orthodontist	23	7.67	3
7	Gondivkar S.M., Sarode S.C., Gadbail A.R., Gondivkar R.S., Chole R., Sarode G.S.	Bibliometric analysis of 100 most cited articles on oral submucous fibrosis	2018	Journal of Oral Pathology and Medicine	23	7.67	3
8	Corbella S., Francetti L., Taschieri S., Weinstein R., Del Fabbro M.	Analysis of the 100 most‐cited articles in periodontology	2017	Journal of Investigative and Clinical Dentistry	21	5.25	6
9	Fardi A., Kodonas K., Lillis T., Veis A.	Top‐cited articles in implant dentistry	2017	International Journal of Oral and Maxillofacial Implants	21	5.25	6
10	Adnan S., Ullah R.	Top‐cited Articles in Regenerative Endodontics: A Bibliometric Analysis	2018	Journal of Endodontics	18	6.00	5
11	Christou P., Antonarakis G.S.	The 100 most‐cited human cleft lip and palate‐related articles published in dentistry, oral surgery, and medicine journals			12	2.00	9

**Citation density**							
**Rank (CY)**	**Author**	**Title**	**Year**	**Journal**	**Citation density**	**Citation**	**Rank (Citation)**
1	Feijoo J.F., Limeres J., Fernández‐Varela M., Ramos I., Diz P.	The 100 most‐cited articles in dentistry	2014	Clinical Oral Investigations	9.57	67	1
2	Pena‐Cristóbal M., Diniz‐Freitas M., Monteiro L., Diz Dios P., Warnakulasuriya S.	The 100 most‐cited articles on oral cancer	2018	Journal of Oral Pathology and Medicine	8.67	26	4
3	Tarazona B., Lucas‐Dominguez R., Paredes‐Gallardo V., Alonso‐Arroyo A., Vidal‐Infer A.	The 100 most‐cited articles in orthodontics: A bibliometric study	2018	Angle Orthodontist	7.67	23	5
4	Gondivkar S.M., Sarode S.C., Gadbail A.R., Gondivkar R.S., Chole R., Sarode G.S.	Bibliometric analysis of 100 most‐cited articles on oral submucous fibrosis	2018	Journal of Oral Pathology and Medicine	7.67	23	5
5	Fardi A., Kodonas K., Gogos C., Economides N.	Top‐cited articles in endodontic journals	2011	Journal of Endodontics	7.50	75	2
6	Ahmad P., Asif J.A., Alam M.K., Slots J.	A bibliometric analysis of Periodontology 2000	2020	Periodontology 2000	7.00	7	8
7	Adnan S., Ullah R.	Top‐cited articles in regenerative endodontics: A bibliometric analysis	2018	Journal of Endodontics	6.00	18	7
8	Garcovich D., Marques Martinez L., Adobes Martin M.	Citation classics in pediatric dentistry: a bibliometric study on the 100 most‐cited articles	2020	European Archives of Pediatric Dentistry	6.00	6	9
9	Ordinola‐Zapata R., Peters O.A., Nagendrababu V., Azevedo B., Dummer P.M.H., Neelakantan P.	What is of interest in Endodontology? A bibliometric review of research published in the International Endodontic Journal and the Journal of Endodontics from 1980 to 2019	2020	International Endodontic Journal	6.00	6	9
10	Corbella S., Francetti L., Taschieri S., Weinstein R., Del Fabbro M.	Analysis of the 100 most‐cited articles in periodontology	2017	Journal of investigative and clinical dentistry	5.25	21	6
11	Fardi A., Kodonas K., Lillis T., Veis A.	Top‐cited articles in implant dentistry	2017	International Journal of Oral and Maxillofacial Implants	5.25	21	6
12	Jafarzadeh H., Sarraf Shirazi A., Andersson L.	The most‐cited articles in dental, oral, and maxillofacial traumatology during 64 years	2015	Dental Traumatology	5.17	31	3

The 100 most‐cited articles in 33 articles were selected based on WoS, Scopus, and PubMed citation data. Two of these 33 articles were dedicated to analyzing the 100 most‐cited publications of the *J Dent Res* and *Periodontol 2000*. Two other articles analyzed the most‐cited 100 articles in different journals, on the subject of *Endodontics* and *Pediatric Dentistry*. Other fields with the most‐cited articles, which were analyzed, are *Core Dental Public Health Journals, Prosthodontic, Periodontal, and Orthodontics*.

**Figure 1:**
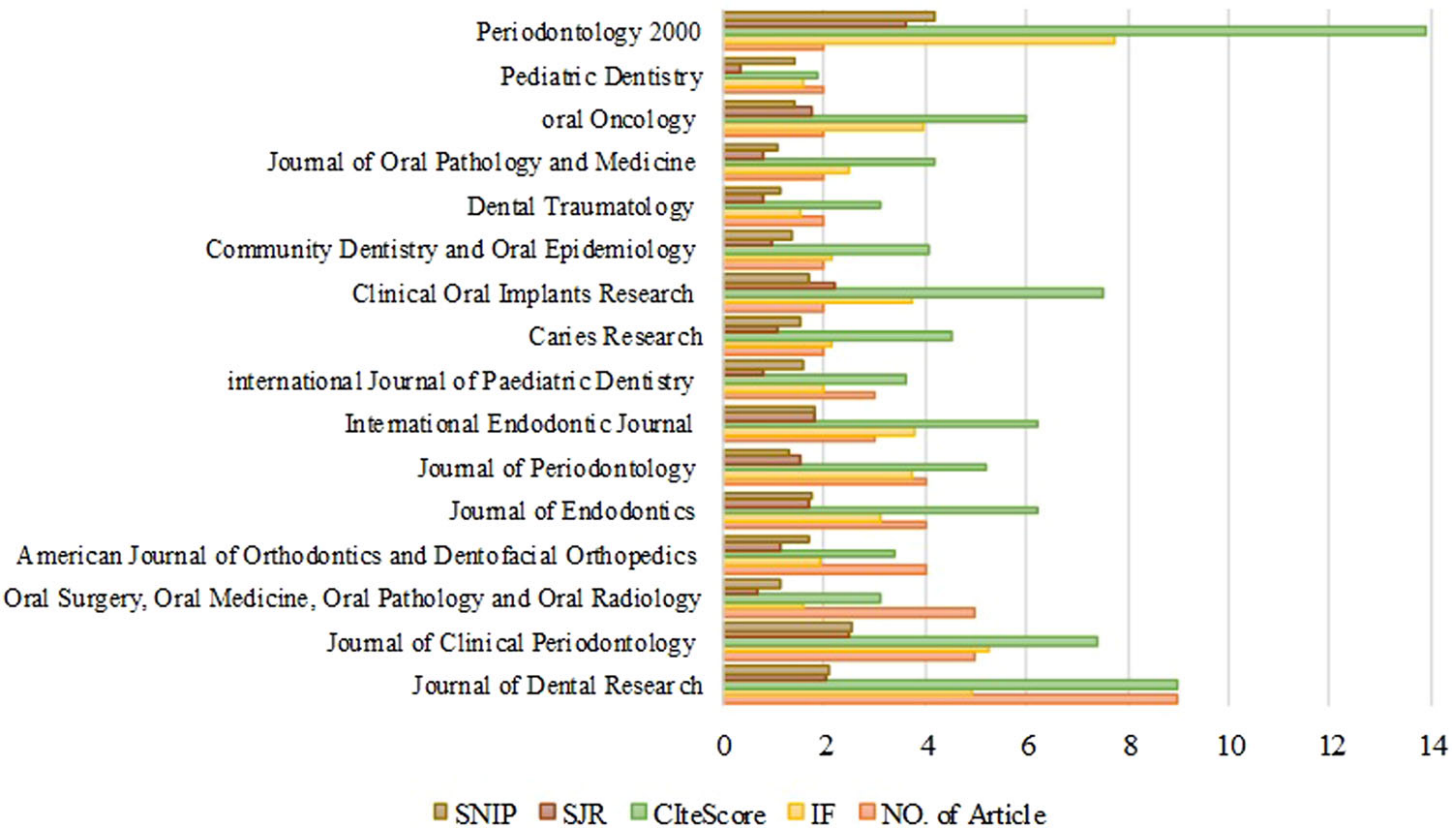
Core Journals in publishing most‐cited articles

Bibliometric analysis, such as the most productive author, journal, institute, and country in publishing most‐cited articles is reported in each of these articles, which are presented in Supporting Information: Appendix 1. As aforementioned, in these 33 articles, the journals that have published the most‐cited articles in the field of dentistry are introduced. Out of 46 journals, three of them were given first to third places due to the high amount of published articles. *J Dent Res*, which was mentioned in nine articles, was introduced as the journal with the publication of articles of highest citation (Figure 1).

All of these journals are indexed in all three databases of WoS, Scopus, and PubMed. In terms of IF, nine journals were Q1, four are Q2, and three of them were Q3, and in terms of CiteScore, all but one was Q1. A quartile in Scopus and WoS is a category of journals that shows their credibility. Each subject category of journals is divided into four quartiles: Q1, Q2, Q3, and Q4. Q1 is occupied by the top 25% of journals in the list; Q2 is occupied by journals in the 25 to 50% group; Q3 is occupied by journals in the 50 to 75% group and Q4 is occupied by journals in the 75 to 100% group.

**Figure 2:**
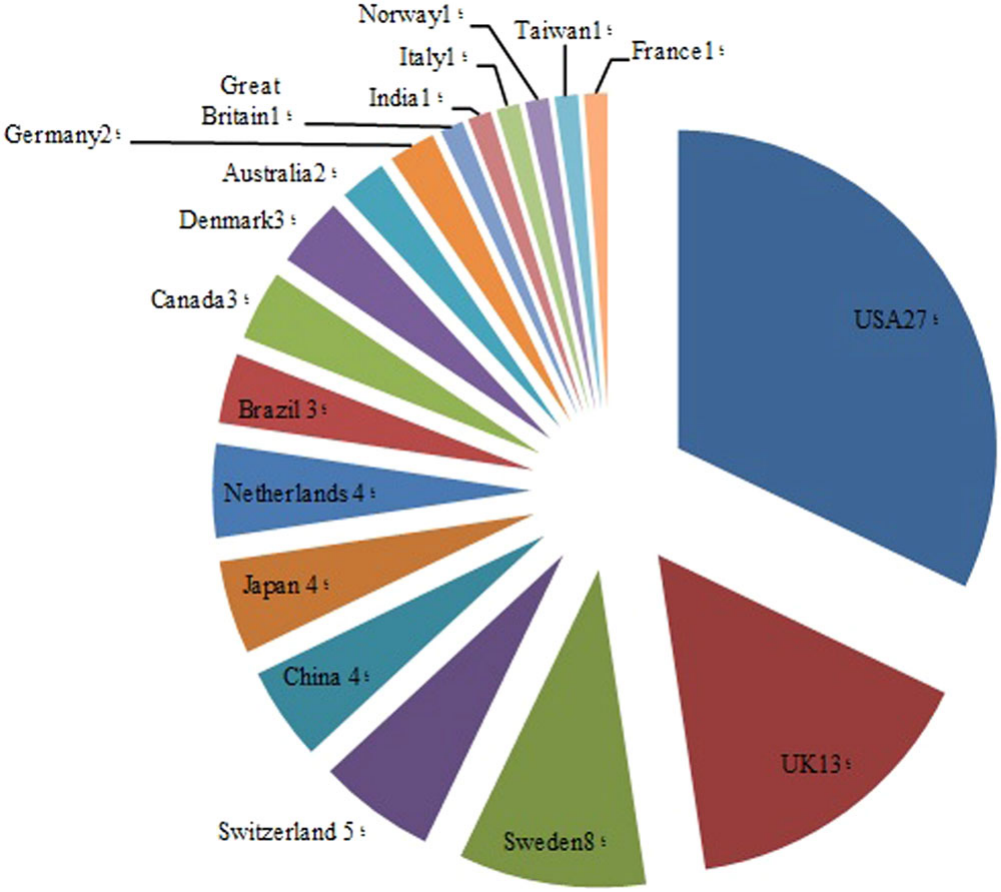
Core countries in publishing most‐cited articles

Also, the most productive country in the publication of most‐cited articles in dentistry was introduced in these 33 articles. The top three countries were extracted (among 18 countries) with the highest count of publications (Figure 2).

According to Figure 2, *The USA* with 27 cases took first place. Considering that in 26 of them, this country was introduced as the first most productive one. In terms of the most productive institutions in the publication of most‐cited articles, the results depict that *the University of Texas* and *the University of Michigan* with six and five cases, respectively, held the first and second places out of 50 introduced institutes. *Loma Linda University* got in third place with four cases. Five institutes with two cases each and 33 institutes with one case were in the following spots of most productive institutes of most‐cited articles in the field of dentistry. In these 33 articles, the topic of 3300 most‐cited articles was also introduced, as well as the introduction of the journals, countries, and core institutes in publishing most‐cited articles (Supporting Information: Appendix 1). The most common research topics in the field of dentistry cover a range of dental public health and pediatric dentistry to adhesive restorative dentistry and implantology. Dental materials, oral medicine, and pathology seem like inseparable topics of common research in dentistry.

## 4 DISCUSSION

The purpose of the study was to analyze the publications, which have studied 3300 top‐cited articles in dentistry. According to the results of the study, two articles published in 2021 are the newest ones. One of them is a study by Mattos, et al., which analyzed the most‐cited articles published in journals related to *Core Dental Public Health*. Regarding the article type, 33 articles were *Cross‐Sectional* and 25 articles were *Review*. From the subject point of view, 84 articles were on *Epidemiology* and 16 were related to *Health Services Research*. The highest amount of top‐cited articles on this subject area was published in *Community Dentistry* and *Oral Epidemiology* (Mattos et al., 2021). Another article published in 2021, analyzes the 100 most‐cited publications in the field of *Oral Medicine and Pathology*. Out of a total of 100 articles, 38 of them concentrated on *Head and Neck Cancer*. *Craniofacial Congenital Anomaly* and *Osteonecrosis* were the following subjects. With respect to the article type, 33 and 26 articles were *Laboratory* and *Descriptive*, respectively (Martelli et al., 2021). The first article in terms of year of publication is in *J Endod* in 2011, analyzing the 100 most‐cited articles on *Endodontic* subjects. The results of this article demonstrate that *J Endod* with 54 articles, *USA* with 52, and *Loma Linda University* with 12 articles, are orderly among core journal, country, and institute in publishing the most‐cited articles. Topics like *Irrigant, Canal Instrumentation, MTA, Leakage, and Endodontic Microbiology* are some of which were addressed a lot in these most‐cited 100 articles (Fardi et al., 2011). This article takes the place of the highest citation (75) out of 33 articles and is in sixth place in terms of citation density (7.5). An article that analyzed the 100 most‐cited publications of 77 journals about *Dentistry, Oral Surgery, and Medicine*, is in the first place of citation density (9.57) and the second place of citations (67). *Socransky SS*. with nine articles and *J Clin Periodontol* with 20 articles are orderly the most productive author and journals in the publication of 100 most‐cited articles. According to the results, 43% of these were concerning *Periodontology* (Feijoo et al., 2014). An article by Pena‐Cristóbal et al, which analyzed 100 most‐cited publications about *Oral Cancer*, is in the second place of citation density (8.67) and the fourth place of citations (26 citation). Some of the most covered topics in the 100 most‐cited articles on *Oral Cancer* were the *Aetiopathogenesis of Oral Cancer, Prognosis, and Treatment*. This article was published in *J Oral Pathol Med* in 2018 (Pena‐Cristóbal et al., 2018).

The results indicated that *Ahmad, Paras* of *AO Research Institute Davos* is the most active author with four articles. Two of his articles focused on analyzing the most‐cited articles of two dental journals. One of the articles analyzed 100 most‐cited articles out of 20,502 publications in the *J Den Res*. This journal is one of the most reputable journals in the oral and dental field which its quartile is *one* based on two indicators of IF (WoS) and CiteScore (Scopus. The results of this study demonstrated that the most‐cited articles published in this journal mainly focused on *Dental Materials, Oral Biology, Periodontology, Restorative Dentistry*, and *Caries*. *Pashley, van Meerbeek, and Lambrechts*, with nine, seven, and six papers, respectively, were the most productive authors and *School of Dentistry, the Catholic University of Belgium* with seven articles, and *the USA* with 52 articles were the most active university and country in producing most‐cited articles in this journal (Ahmad et al., 2019). Another article analyzed the 100 most‐cited articles in the *Periodontol 2000* journal. This journal is one of the most reputable journals of Wiley publications with IF = 7.718 and H‐Index = 115. The quartile of this journal is also *one*. In this article, 100 most‐cited articles from this journal between 1993 and 2019 were analyzed. Among the major topics discussed in these articles were *Microbial Etiology of Periodontal Diseases, Pathogenesis of Periodontal Diseases, Periodontium, Tissue Engineering, and Periodontal Regeneration*. The *USA* with 51 articles and *Forsyth Institute* were reported as the most productive country and institute in the publication of most‐cited articles (Ahmad, Asif, et al., 2020).

In another article, out of 4418 articles related to *Systemic Manifestations of Periodontal Disease*, 100 most‐cited ones were chosen for analysis. The results showed that *J Periodontol* with 23 articles, *the USA* with 61 articles, and *Genco* with 13 articles were respectively the core journal, country, and author in producing the most‐cited articles. *Association of Periodontal Disease with Cardiovascular Diseases* and *Diabetes Mellitus* with 31 and 29 articles respectively were among the most popular topics of the 100 most‐cited articles of analysis (Ahmad, Arshad, et al., 2020). In another study, out of 31,584 articles indexed in WoS related to *Dental Caries*, 100 most‐cited articles were analyzed. *J Dent Res* and *Caries Res* with 19 and 17 articles each, *the USA* with 45 articles, and the *University of Gothenburg* from *Sweden* with seven articles were respectively the most productive journal, country, and institute in the publication of most‐cited articles on this topic (Arshad et al., 2020).

Out of these 33 articles, two articles analyzed 100 most‐cited articles in a dental field journal, including the *J Dent Res* (Ahmad et al., 2019) and *Periodontol 2000* (Ahmad, Asif, et al., 2020). Some studies also focused on the 100 most‐cited publications of several journals on one topic. These include four journals in the *Pediatric Dentistry* field (Garcovich et al., 2020) and two *Endodontic Fields* (Ordinola‐Zapata et al., 2020). Other subjects of the most‐cited publications that were analyzed in these journals are *Core Dental Public Health Journals* (Mattos et al., 2021), *Prosthodontic* (Praveen et al., 2020), *Periodontal* (Chiang et al., 2018), and *Orthodontics* (Hui et al., 2013).

Five articles also analyzed the publications in journals of oral and dental categories in WoS and Scopus (Fardi et al., 2011; Feijoo et al., 2014; Gogos et al., 2020; Gonçalves et al., 2019; Perazzo et al., 2019). Gogos et al. focused on analyzing 100 most‐cited articles of *Systematic Review and Meta‐Analysis* in 91 journals in *Dentistry, Oral Surgery, and Medicine* categories. The subjects mentioned in these most‐cited articles were *Implants, Periodontology, and Prosthetics* which were mentioned in 34, 23, and 12 articles, respectively. *Zwahlen and Pjetursson* with 14 and 13 articles, respectively *Clinical Oral Implants Research* and *Journal of Clinical Periodontology* with 22 and 20 articles each were assigned the most productive authors and journals for publishing the most‐cited systematic review articles in the field of dentistry (Gogos et al., 2020). One of these studies also analyzed the most‐cited articles in the dental field of a specific country. Gonçalves et al. analyzed the most‐cited articles published between 1996 and 2017 in 178 dental journals, which had at least one Brazilian author (Gonçalves et al., 2019). Other studies, covered articles of top‐citation related to different topics in the oral and dental field: *Dental Public Health*, *Oral Medicine and Pathology*, *Dental Stem Cells*, *Prosthodontic*, *Early Childhood Caries*, *Oral Potentially Malignant Disorders*, *Oral Lichen Planus*, *Periodontology*, *Cariology*, *Dental Caries*, *Fluoride in the Context of Oral Health*, *Pediatric Dentistry*, *Oral Leukoplakia*, *Orthognathic Surgery*, *Oral Cancer*, *Oral Submucous Fibrosis*, *Periodontal*, *Regenerative Endodontics*, *Implant*, *Oral, and Maxillofacial Trauma*, *Cleft Lip* and/or *Palate, Orthodontics, Endodontic*.

The results of the bibliographic analysis, such as the most productive author, journal, institutes, and the country in each article are presented in Supporting Information: Appendix 1. All of these 16 journals that were mentioned in more than two publications as the most productive journals of 100 most‐cited articles on different subjects were indexed in all three databases of Scopus, PubMed, and WoS. Meanwhile, with nine times of introduction in different journals as the publisher of the highest amount of most‐cited articles in this area, *Dent Res J*,  *J Clin Periodontol*, and *Oral Surg Oral Med Oral Pathol Oral Radiol* were in the second place with five times of introduction in journals. As for the IF indicator, nine journals were Q1 and as for the CiteScore, all but one were Q1. Out of the 30 articles, which were only mentioned in one article as the core journal, only 23 were focused on the dental fields. Seven journals of *Cancer*, *Clin Cancer Res*, *Int J Cancer, J Natl Cancer Inst, Nat Genet, NEJM, and Lancet* from other nondental fields were all Q1. Out of these seven journals, four were in the field of *cancer*. The country with the highest production of oral and dental articles is *the USA*. So in 26 papers, the USA ranked first in terms of producing 100 most‐cited articles.

## 5 CONCLUSION

Analyzing studies that focused on the bibliometric analysis of 100 most‐cited articles of different scopes can provide valuable information for the researchers in these subject areas. Researchers and specialists can get familiar with the most productive author, journal, country, and different institutes for publishing high‐quality articles in the field of oral and dental subjects by the means of the results of this article. Furthermore, the results of this study ranked the most‐cited article topics, which are of interest for publication, demonstrating the future map road oral and dental research. One of the items that was mentioned in these articles is the topic most discussed in the most‐cited articles of each subject field. Familiarity with these topics can be used to determine the research priorities; Because these topics have been able to receive more citations. From another point of view, the results of this study shed light on the topics that were never subject to high citation analysis. It is recommended that analysis studies be carried out about the 100 most‐cited articles in this area.

## AUTHOR CONTRIBUTIONS

All authors gave their final approval and agree to be accountable for all aspects of the work. Ghazaleh Daryakenari: Contributed to data acquisition and interpretation, drafted, and critically revised the manuscript. Zahra Batooli: Contributed to conception, design, data acquisition and interpretation, performed all statistical analyses, drafted, and critically revised the manuscript.

## CONFLICT OF INTEREST

The authors declare no conflict of interest.

## ETHICS STATEMENT

This study was approved by the Kashan University of Medical Sciences Ethics Committee (approval no. IR.KAUMS.NUHEPM.REC.1400.023). Accordingly, there is no need for informed consent from the study populations for bibliometric analysis.

## Supporting information

Supporting information.

## Data Availability

The article submitted is a bibliometric article.

## References

[cre2633-bib-0001] Ahmad, P. , Alam, M. K. , Jakubovics, N. S. , Schwendicke, F. , & Asif, J. A. (2019). 100 years of the Journal of Dental Research: A bibliometric analysis. Journal of Dental Research, 98(13), 1425–1436. 31746684 10.1177/0022034519880544

[cre2633-bib-0002] Ahmad, P. , Arshad, A. I. , Bella, E. D. , Khurshid, Z. , & Stoddart, M. (2020). Systemic manifestations of the periodontal disease: A bibliometric review. Molecules, 25(4508), 1–17. 10.3390/molecules25194508 PMC7582719 33019648

[cre2633-bib-0003] Ahmad, P. , Asif, J. A. , Alam, M. K. , & Slots, J. (2020). A bibliometric analysis of periodontology 2000. Periodontology, 82(1), 286–297. 10.1111/prd.12328 31850637

[cre2633-bib-0004] Aminpour, F. , Kabiri, P. , & Naji, H. (2008). Isfahan University of Medical Sciences: Two decades of scientific achievements. Iranian Journal of Medical Education. 8(1), 164–173.

[cre2633-bib-0005] Arshad, A. I. , Ahmad, P. , Dummer, P. M. H. , Alam, M. K. , Asif, J. A. , Mahmood, Z. , Rahman, N. A. , & Mamat, N. (2020). Citation classics on dental caries: A systematic review. European Journal of Dentistry, 14(1), 128–143. 32189321 10.1055/s-0040-1703419 PMC7069738

[cre2633-bib-0006] Baldiotti, A. L. P. , Amaral‐Freitas, G. , Barcelos, J. F. , Freire‐Maia, J. , Perazzo, M. F. , Freire‐Maia, F. B. , Paiva, S. M. , Ferreira, F. M. , & Martins‐Júnior, P. A. (2021). The top 100 most‐cited papers in cariology: A bibliometric analysis. Caries Research, 55(1), 32–40. 33341798 10.1159/000509862

[cre2633-bib-0007] Ball, E. , McLoughlin, M. , & Darvill, A. (2011). Plethora or paucity: A systematic search and bibliometric study of the application and design of qualitative methods in nursing research 2008–2010. Nurse Education Today, 31(3), 299–303. 21295895 10.1016/j.nedt.2010.12.002

[cre2633-bib-0008] Bazrafshan, A. , & Mostafavi, E. (2011). A scientometric overview of 36 years of scientific productivity by Pasteur Institute of Iran in ISI SCIE. Journal of Health Administration, 14(45), 7–10.

[cre2633-bib-0009] Bornmann, L. , & Daniel, H. D. (2008). What do citation counts measure? A review of studies on citing behavior. Journal of Documentation, 64(1), 45–80.

[cre2633-bib-0010] Chang, C. Y. , Gau, M. L. , Tang, K. Y. , & Hwang, G. J. (2021). Directions of the 100 most cited nursing student education research: A bibliometric and co‐citation network analysis. Nurse Education Today, 96 104645. 10.1016/j.nedt.2020.104645 33166794

[cre2633-bib-0011] Chiang, H. S. , Huang, R. Y. , Weng, P. W. , Mau, L. P. , Su, C. C. , Tsai, Y. W. C. , Wu, Y. C. , Chung, C. H. , Shieh, Y. S. , & Cheng, W. C. (2018). Increasing prominence of implantology research: A chronological trend analysis of 100 top‐cited articles in periodontal journals. European Journal of Oral Implantology, 11(1), 97–110. 29557404

[cre2633-bib-0012] Das, J. P. , Aherne, E. , & Kavanagh, E. (2019). Imaging of the spine: A bibliometric analysis of the 100 most‐cited articles. Spine, 44(22), 1593–1598. 31689254 10.1097/BRS.0000000000003131

[cre2633-bib-0013] Elarjani, T. , Almutairi, O. T. , Alhussinan, M. , Alturkistani, A. , Alotaibi, F. S. , Bafaquh, M. , & Alotaibi, F. E. (2021). Bibliometric analysis of the top 100 most cited articles on craniosynostosis. Child's Nervous System, 37(2), 587–597. 10.1007/s00381-020-04858-2 32780272

[cre2633-bib-0014] Elarjani, T. , Almutairi, O. T. , Alhussinan, M. , Alzhrani, G. , Alotaibi, F. E. , Bafaquh, M. , Orz, Y. , AlYamany, M. , & Alturki, A. Y. (2021). Bibliometric analysis of the top 100 most cited articles on cerebral vasospasm. World Neurosurgery, 145, e68–e82. 10.1016/j.wneu.2020.09.099 32980568

[cre2633-bib-0015] Fardi, A. , Kodonas, K. , Gogos, C. , & Economides, N. (2011). Top‐cited articles in endodontic journals. Journal of Endodontics, 37(9), 1183–1190. 21846531 10.1016/j.joen.2011.05.037

[cre2633-bib-0016] Feijoo, J. F. , Limeres, J. , Fernández‐Varela, M. , Ramos, I. , & Diz, P. (2014). The 100 most cited articles in dentistry. Clinical Oral Investigations, 18(3), 699–706. 23771182 10.1007/s00784-013-1017-0

[cre2633-bib-0017] Gao, Q. , Zhang, C. , Wang, J. , Wei, Q. , Wei, Q. , Miyamoto, A. , Zhu, S. , & He, C. (2020). The top 100 highly cited articles on osteoporosis from 1990 to 2019: A bibliometric and visualized analysis. Arch Osteoporosis, 15(144), 1–11. 10.1007/s11657-020-0705-z 32935223

[cre2633-bib-0018] Garcovich, D. , Marques Martinez, L. , & Adobes Martin, M. (2020). Citation classics in paediatric dentistry: A bibliometric study on the 100 most‐cited articles. European Archives of Paediatric Dentistry, 21(2), 249–261. 31562594 10.1007/s40368-019-00483-z

[cre2633-bib-0019] Gogos, C. , Kodonas, K. , Fardi, A. , & Economides, N. (2020). Top 100 cited systematic reviews and meta‐analyses in dentistry. Acta Odontologica Scandinavica, 78(2), 87–97. 31418611 10.1080/00016357.2019.1653495

[cre2633-bib-0020] Gonçalves, A. P. , Plá, A. L. , Rodolfo, B. , Nahsan, F. P. , Correa, M. B. , & de Moraes, R. R. (2019). Top‐10 0 most cited dental articles with authors from Brazil. Brazilian Dental Journal, 30(2), 96–105. 30970066 10.1590/0103-6440201902529

[cre2633-bib-0021] Gupta, A. , Kennedy, B. , Meriwether, K. V. , Francis, S. L. , Cardenas‐Trowers, O. , & Stewart, J. R. (2020). Citation classics: The 100 most cited articles in urogynecology. International Urogynecology Journal, 31(2), 249–266. 31309245 10.1007/s00192-019-04021-9

[cre2633-bib-0022] Hui, J. , Han, Z. , Geng, G. , Yan, W. , & Shao, P. (2013). The 100 top‐cited articles in orthodontics from 1975 to 2011. Angle Orthodontist, 83(3), 491–499. 23050741 10.2319/040512-284.1 PMC8763075

[cre2633-bib-0023] Joyce, C. W. , Kelly, J. C. , & Carroll, S. M. (2014). The 100 top‐cited classic papers in hand surgery. Journal of Plastic Surgery and Hand Surgery, 48(4), 227–233. 24063628 10.3109/2000656X.2013.840640

[cre2633-bib-0024] Karydakis, P. , Giakoumettis, D. , & Themistocleous, M. (2020). The 100 most cited papers about pediatric traumatic brain injury: A bibliometric analysis. Irish Journal of Medical Science, 189(1), 315–325. 31418153 10.1007/s11845-019-02085-6

[cre2633-bib-0025] Koh, B. , Banu, R. , & Sabanayagam, C. (2020). The 100 most cited articles in ophthalmology in Asia. Asia‐Pacific Journal of Ophthalmology, 9(5), 379–397. 32956190 10.1097/APO.0000000000000325

[cre2633-bib-0026] Mainwaring, A. , Bullock, N. , Ellul, T. , Hughes, O. , & Featherstone, J. (2020). The top 100 most cited manuscripts in bladder cancer: A bibliometric analysis (review article). International Journal of Surgery, 75, 130–138. 31991242 10.1016/j.ijsu.2020.01.128

[cre2633-bib-0027] Martelli, A. J. , Martelli, R. A. M. , Martelli, D. R. B. , das Neves, L. T. , & Martelli Junior, H. (2021). The 100 most‐cited papers in oral medicine and pathology. Brazilian Oral Research, 35, e0201‐14. 10.1590/1807-3107bor-2021.vol35.0020 33331410

[cre2633-bib-0028] Martinez‐Perez, R. , Ung, T. H. , & Youssef, A. S. (2021). The 100 most‐cited articles on vestibular schwannoma: Historical perspectives, current limitations, and future research directions. Neurosurgical Review, 44, 2965–2975. 10.1007/s10143-021-01487-4 33523339

[cre2633-bib-0029] Mattos, F. D. F. , Perazzo, M. F. , Vargas‐Ferreira, F. , Martins‐Júnior, P. A. , & Paiva, S. M. (2021). Top 100 most‐cited papers in core dental public health journals: Bibliometric analysis. Community Dentistry and Oral Epidemiology, 49(1), 40–46. 32935344 10.1111/cdoe.12572

[cre2633-bib-0030] Moed, H. F. (2009). New developments in the use of citation analysis in research evaluation. Archivum Immunologiae et Therapiae Experimentalis, 57(1), 13–18. 19219533 10.1007/s00005-009-0001-5

[cre2633-bib-0031] Ordinola‐Zapata, R. , Peters, O. A. , Nagendrababu, V. , Azevedo, B. , Dummer, P. M. H. , & Neelakantan, P. (2020). What is of interest in endodontology? A bibliometric review of research published in the International Endodontic Journal and the Journal of Endodontics from 1980 to 2019. International Endodontic Journal, 53(1), 36–52. 31454086 10.1111/iej.13210

[cre2633-bib-0032] Paladugu, R. , Schein, M. , Gardezi, S. , & Wise, L. (2002). One hundred citation classics in general surgical journals. World Journal of Surgery, 26(9), 1099–1105. 12209239 10.1007/s00268-002-6376-7

[cre2633-bib-0033] Pawson, R. , Greenhalgh, T. , Harvey, G. , & Walshe, K. (2004). Realist synthesis: An introduction. ESRC Research Methods Programme, University of Manchester. https://www.betterevaluation.org/sites/default/files/RMPmethods2.pdf

[cre2633-bib-0034] Pena‐Cristóbal, M. , Diniz‐Freitas, M. , Monteiro, L. , Diz Dios, P. , & Warnakulasuriya, S. (2018). The 100 most cited articles on oral cancer. Journal of Oral Pathology and Medicine, 47(4), 333–344. 29380894 10.1111/jop.12686

[cre2633-bib-0035] Perazzo, M. F. , Otoni, A. L. C. , Costa, M. S. , Granville‐Granville, A. F. , Paiva, S. M. , & Martins‐Júnior, P. A. (2019). The top 100 most‐cited papers in Paediatric Dentistry Journals: A bibliometric analysis. International Journal of Paediatric Dentistry, 29(6), 692–711. 31325392 10.1111/ipd.12563

[cre2633-bib-0036] Praveen, G. , Chaithanya, R. , Alla, R. K. , Shammas, M. , Abdurahiman, V. T. , & Anitha, A. (2020). The 100 most cited articles in prosthodontic journals: A bibliometric analysis of articles published between 1951 and 2019. Journal of Prosthetic Dentistry, 123(5), 724–730. 31474409 10.1016/j.prosdent.2019.05.014

[cre2633-bib-0037] Shuaib, W. , Khan, M. S. , Shahid, H. , Valdes, E. A. , & Alweis, R. (2015). Bibliometric analysis of the top 100 cited cardiovascular articles. The American Journal of Cardiology, 115(7), 972–981. 25670637 10.1016/j.amjcard.2015.01.029

[cre2633-bib-0038] van Hooft, S. M. , Been‐Dahmen, J. M. , Ista, E. , van Staa, A. , & Boeije, H. R. (2017). A realist review: What do nurse‐led self‐management interventions achieve for outpatients with a chronic condition? Journal of Advanced Nursing, 73(6), 1255–1271. 27754557 10.1111/jan.13189

[cre2633-bib-0039] Zhong, Q. , Li, B. H. , Zhu, Q. Q. , Zhang, Z. M. , Zou, Z. H. , & Jin, Y. H. (2019). The top 100 highly cited original articles on immunotherapy for childhood leukemia. Frontiers in Pharmacology, 10(1100), 1–9. 31611792 10.3389/fphar.2019.01100 PMC6769078

