# Supplementary material for: A bibliometric and subject analysis of 3300 most‐cited articles in dentistry
Source: Clin Exp Dent Res. 2022 Aug 7;8(5):1302–10. doi: 10.1002/cre2.633 (PMC9562829; doi:10.1002/cre2.633)
Supplement: Supplementary file 1 — Supporting information. [file CRE2-8-1302-s001.doc]

**Appendix 1. Analysis of 33 articles**

| **Author** | **Database** | **Subject** | **Most Common Research Topic** | **Prominent Authors** | **Prominent Journal** | **Prominent Country** | **Prominent Instituation** | **Article Type/Study Design/ Level Of Evidence** |
| --- | --- | --- | --- | --- | --- | --- | --- | --- |
|  | WoS | Dental Public Health | epidemiology. | Locker D, Petersen PE, Sheiham A | Community Dentistry, Oral Epidemiology | UK, USA,,Canada | University Of Toronto, University College London And Royal Dental College | Cross-sectional and Nonsystematic review |
|  | WoS | Oral Medicine And Pathology | head and neck cancer, craniofacial congenital anomalies, and osteonecrosis |  | The New England Journal of Medicine, , Nature Genetics, lancet | USA, UK,France |  | Laboratory, Descriptive, Randomized; Few studies: clinical, retrospective, or comparative studies, or case-reports |
|  | Unknown | Dental Stem Cells |  |  | Journal of Dental Research | USA, China | Seoul National University | original and review |
|  | WoS | Prosthodontic | dental implants, composite resins and ceramics, TMJ, CD, posts FPD/crowns, occlusion | Albrektsson T, Zarb GA | Journal of Prosthetic Dentistry , International Journal of Prosthodontics | USA, Sweden, Canada |  | reviews, experimental |
|  | Scopus | Early Childhood Caries | aetiology/associated factors, preventive measures/treatment, prevalence | Hughes CV, Ismail AI, Kanasi E, Tanner ACR, Vann WF | Community Dentistry and Oral Epidemiology journal, Pediatric Dentistry, Journal of Dental Research | USA, Australia, Brazil, Canada | University of Queensland, Forsyth Institute, Harvard University, New York University, University of North Carolina, and the University of Michigan. | original, review, Cross-sectional, expert opinion/narrative reviews and cohort |
|  | Scopus | Oral Potentially Malignant Disorders | Molecular markers/targets, Treatment (Chemoprevention, Vitamin A, retinoid and betacarotene, Natural products, eg. Curcumin and tea), Early detection and diagnosis of oral cancer, Follow-up outcome, Etiology and risk factors (Betel quid chewing, tobacco and alcohol use, etc, Human papillomavirus), Narrative review of comprehensive knowledge, Epidemiology, Utility and subjectivity of oral dysplasia, Animal model of oral carcinogenesis | Hong WK, van der, Lippman S | Oral Surg Oral Med Oral Pathol, Oral Radiol, Oral Oncol | USA, UK, Netherlands | M.D. Anderson Cancer Center | primary research, narrative review, systematic review or meta-analysis;  higher evidence level: cohort studies, Systematic reviews, randomized controlled trial but the large majority were considered lower level |
|  | Scopus | Oral Lichen Planus | Malignant transformation , etiology and etiopathogenesis , treatment, epidemiology,lichenoid lesions or reactions, and molecular markers/targets of oral carcinogenesis | van der Waal I., Scully C, Carrozzo M. | Journal of Oral Pathology and Medicine, Oral Surgery ,Oral Medicine, Oral Pathology ,Oral Radiology | USA, UK, Italy, Netherlands | Academic Centre for Dentistry Amsterdam, School of Dentistry,  University of California, Eastman Dental Institute, and School of Medicine and Dentistry, University of Turin | primary research, narrative review, systematic review or meta-analysis  case report ( level 4), cohort studies ( level 3), RCT article (level 2), systematic review (level 1) |
|  | WoS | Periodontal Disease | cardiovascular diseases, diabetes mellitus, Collective systemic manifestations, pregnancy-related  manifestations ,rheumatic ,pulmonary ,cerebrovascular diseases ,cancer | Genco RG,, Beck JD, Offenbacher S, Taylor GW, Tonetti MS, Suvan J | Journal of Periodontology, Annals of Periodontology, Journal of Clinical Periodontology | USA, UK | School of Dental Medicine, University, School of Dentistry, University,  College of Dental Medicine | clinical trials, literature review, randomized controlled trials, case-control, systematic review, meta-analysis  Level V, III , I,II , IV evidence |
|  | WoS | Periodontology | microbial etiology of periodontal diseases, pathogenesis of periodontal diseases, periodontium, tissue engineering and periodontal regeneration, epidemiology of periodontal diseases, risk factors for periodontal diseases, dental implantology, antibiotics in periodontology, miscellaneous (chlorhexidine or titanium), nonsurgical periodontal therapy, peri‐implantitis, systemic diseases and periodontal health, and dental biofilm | P.M. Bartold, A.D. Haffajee/ S.S. Socransky, J.M. Albandar |  | USA, Switzerland, Australia | Forsyth Institute, University of Queensland, University of Bern |  |
|  | WoS | Cariology | etiology/pathogenesis, prevention | Featherstone J.D.B., Axelsson P, Lindhe J. | Dental Research | USA, Sweden, Japan | University of Michigan, University of Helsinki | Literature reviews, laboratorial |
|  | WoS | Dental Caries | dental caries, Streptococcus mutans , fluoride, dentin, lactobacillus, saliv, Actinomyces, dental plaqu, and gingivitis | J.D. Featherstone, P. Axelsson, J. Lindhe | Journal of Dental Research, Caries Research, Archives of Oral Biology | USA, Sweden, UK | University of Gothenburg, Gothenburg, Sweden, School of Dentistry, University of Michigan, Ann Arbor | review, clinical, laboratory, animal, new classification/ tool/technique, cohort studies;  Level V, IV, III, II, and I evidence |
|  | WoS | Dentistry | Implants,Periodontology,Prosthetics,Operative dentistry,Oral conditions,Materials, Endodontics, Orthodontics, Temporomandibular Disorders, Dental agenesis, Dental health education, Radiology, Recommendations | Zwahlen M, Pjetursson BE, Lang NP | Clinical Oral Implants Research, Journal of Clinical Periodontology | USA, Switzerland, UK, Netherlands | University of Zurich, Radboud University Nijmegen, University of Bern |  |
|  | WoS | Paediatric Dentistry | Caries/ECC (early childhood caries), Pulp therapy, Dental trauma, Dental anomalies, OHRQL, MIH, Behaviour management, Prevention, Dental erosion, Orthodontics/occlusion, Access to dental, Dental age, Biology, Stem cells,Child abuse, Anaesthesia or sedation,Systemic interaction,Oral pathology, Dental materials, Restorative treatment | Feigal RJ, Holan G | Paediatric dentistry, International journal of paediatric dentistry | USA, UK | University of Michigan, Hebrew, University of Jerusalem | Literature review, Cross-sectional, Retrospective, Cohort, Observational/longitudinal, CCT/RCT, Case–control,In vitro, Guideline/policy, Case report, Conference paper, Systematic review |
|  | WoS | Endodontic | between 1980 and 1999: host immune response (microbiology, pathology)  During 2000–2009: research on either pulp therapy, biocompatibility, immunology or regenerative endodontics  From 2010 to 2019: . CBCT imaging and tomography appears for the first time within the most used terms. Research on dental materials was represented by the term bond strength. Four research themes were clearly differentiated: pulp biology, nickel-titanium instruments, local anaesthesia and physical or biological properties of dental materials including antimicrobials. One cluster was associated with terms  linked to several aspects of clinical practice such as CBCT, review, case report, vertical root fracture, diagnostic accuracy, prevalence, prospective study, autotransplantation, canal morphology, electronic apex locator, external cervical resorption, periapical lesion, among others |  |  | USA, Brazil, UK | United States Army, Loma Linda, University, University College London and University of Sao Paulo | During the last 10 years, an increase in the number of reviews and papers on cone beam computed tomography occurred. |
|  | WoS | Endodontics | Endodontic microbiology, Regenerative endodontics, MTA, Canal instrumentation, Restoration, Success and failure studies, Irrigation 8Intracanal dressing, Vital pulp therapy, Dentinogenesis,Leakage, Obturation, Traumatic dental injuries, Root canal anatomy, Dentin hypersensitivity, Pulp neurophysiology, Pulpal diseases, Radiology, Dentin microstructure, Root resorption | Torabinejad M, Sundqvist G, Shi S | Journal of Endodontics, International Endodontic Journal, Dental Traumatology Oral Surgery Oral Medicine Oral Pathology Oral Radiology and Endodontology | USA, Sweden, Switzerland | Umeå University, Loma Linda University,  University of Zurich | basic, review, clinical, guidelines, new technique  study design: Non-randomized experimental, Cross-sectional, Retrospective cohort, Case series, Inception cohort, Case–control, Case report  Half of the clinical articles: level III evidence |
|  | Scopus | Fluoride In The Context Of Oral Health |  | Klimek J, Ganss C,  Cury JA | Caries Research, Journal of Dental Research, American Journal of Orthodontics and Dentofacial Orthopaedics, European Journal of Oral Sciences, Journal of the American Dental Association | USA, Germany, UK | University of Oslo, University of California, San Francisco, the Justus Liebig University, Academic Centre for Dentistry Amsterdam | Original (In vitro, Original, Randomized control trial, Cross-sectional, Animal, Qausi experimental, Case-control); Reviews ( Narrative review, Systematic review, Cochrane review, Meta-analysis) |
|  | WoS | Paediatric Dentistry | Cariology, Dental growth and development, Behavioural and Epidemiological Science, Paediatric endodontics, Dental materials, Dental trauma, Others | Messer LB, van Amerongen WE, Weerheijm KL | International Journal of Paediatric Dentistry, international Journal of Paediatric Dentistry, Journal of Dentistry for Children, Pediatric Dentistry | USA, Brazil |  | cross-sectional design, Literature review, Laboratorial, Clinical trial, Longitudinal, Retrospective, Randomized clinical trial, Case‐control, Systematic review, Retrospective, Case series, Case report |
|  | Scopus | Oral Leukoplakia | chemopreventive drugs, etiology and risk factors of oral cancer and premalignant lesions including OL, epidemiology of oral mucosal diseases including OL, follow-up outcome studies, molecular markers/targets of oral carcinogenesis | Hong W.K., Lippman S, Pindborg J.J. | Journal of Oral Pathology and Medicine, Oral Surgery, Oral Medicine, Oral Pathology, Oral Radiology | USA, UK, Danmark, China | M.D. Anderson Cancer Center, University of Texas, Houston; National Cancer Institute, Bethesda, Maryland; King’s College Dental Institute, Denmark Hill Campus, London; Academic Centre for Dentistry Amsterdam (ACTA), Amsterdam | Systematic reviews/ meta-analysis, randomized controlled trial  primary research articles, narrative review, systematic review or meta-analysis.  3 systematic review or meta-analysis: level 1; 7 RCT: level 2; 20 cohort studies: level 3; 70 articles: lower level |
|  | Scopus | Dentistry (Brazil) | Dental Materials, Endodontics, Periodontology, Oral Biology, Oral and Maxillofacial, Surgery, Implantology, Oral Radiology, Restorative Dentistry, Pediatric Dentistry, Oral Pathology, Cariology Public Health | In total, 70% of the papers had first authors  based in Brazil, but 40% had corresponding authors affiliated to international | J Dent Res, J Endod, Int Endod J | Southeast and South were the regions of Brazil with most presence of coauthors | University of São Paulo (USP), University of Campinas (UNICAMP), Estácio de Sá University | original, reviews |
|  | Scopus | Dentistry, Oral Surgery, And Medicine | materials science, oral biology, periodontology, restorative dentistry, caries, regenerative dentistry, implant dentistry, bone morphology/histology, oral hygiene, oral medicine/pathology, rosthodontics,orthodontics, behavior management, endodontics | Pashley, van Meerbeek, Lambrechts |  | USA, Netherlans Japan | School of Dentistry, Catholic University of Leuven, Belgium; Academic Center for Dentistry Amsterdam, University of Amsterdam, Netherlands; Forsyth Dental Center, United Statesl; National Institute of Dental And Craniofacial Research, National Institutes of Health, United States | Research Reports–Biomaterials & Bioengineering, Reviews (systematic reviews), Research-Reports Clinical, Research Reports–Biological |
|  | WoS | orthodontics | Mini-implants, Biomechanics and biology, Root resoption, Demineralizacion, New technologies, Wire, Orthognathic surgery, Injuries/discomfort, Adhesion, Assessing index, Airway, Cleft lip and/or palate, Asymmetries, Cephalometry, Class II treatment, Corticotomies, Esthetic smile, Facial stetic, Friction, Genetics, Growth and development, Maxillary canines, Mixed dentition treatment, Perception esthetics, Stability orthodontic treatment, Temporomandibular joint, Transplanted teeth | Takano-Yamamoto, T, Deguchi, T, Zachrisson, BU | American Journal of Orthodontics and Dentofacial Orthopedics, Angle Orthodontist, Journal of Dental Research | USA, Norway, Japan | University of Oslo, The Ohio State University, University of North Carolina | Clinical, Review, Basic |
|  | WoS, Pubmed, Google scholar | Orthognathic Surgery | diagnosis,fixation/stability, virtual planning/CT analysis,complications,  technique, airway/OSA, cephalometric analysis, psychosocial, anatomy and physiology | 70%  North America and  30% international | Journal specialty:  Oral and maxillofacial surgery, Otolaryngology, Orthodontics, Plastic and reconstructive surgery,Other medical/surgical specialty |  |  | Study designs: cohort studies, case report/ series, review, descriptive studies, experimental, technique, clinical trials, systematic review |
|  | WoS | Oral Cancer | aetiopathogenesis of oral cancer , prognosis , treatment  most widely studied risk factor was tobacco, including chewing  tobacco and other products, followed by HPV and alcohol. | Blot, WJ, Franceschi, S, Fraumeni, JF, Winn DM | Cancer, Clinical Cancer Research, Journal of the National Cancer Institute, International Journal of Cancer | USA, Uk, China | M.D. Anderson Cancer Center, University  of Texas; Houston; National Cancer Institute, Bethesda; Maryland Memorial Sloan-Kettering Cancer Center, New York; University of California, San Francisco | primary, clinical, basic, epidemiological, narrative reviews, meta-analyses  The majority of studies were classified as evidence level 4, only 2 articles (the meta-analyses) were considered level 1, The 17 narrative reviews were classified as level 5. |
|  | Scopus | Oral Submucous Fibrosis |  | Gupta P.C., Pindborg J.J., Daftary D.K. | Journal al of Oral Pathology and Medicine, oral Oncology, community Dentistry and Oral Epidemiology | India, Taiwan, UK | Basic Dental Research Unit, Tata; Institute of Fundamental Research, Mumbai ; Eastman Dental Hospital, London; National Taiwan University, Taipei | primary (clinical, epidemiological, basic); review articles  Most of the clinical studies were classified as evidence level 4 and 28 review articles were classified as level 5 |
|  | wos | Periodontal | inflammatory periodontal disease |  |  |  | University of Gothenburg (Sweden), Forsyth Dental Center (USA) | original |
|  | WoS | Regenerative Endodontics | Regenerative endodontics, Revascularization, Stem cells, Dental pulp stem cells, Pulp regeneration, Tissue engineering, Immature teeth, Regeneration, Pulp revascularization Apexification, mineral trioxide aggregate | Mahmoud Torabinejad, Thibodeau Blayne, Nosrat Ali | Journal of Endodontics, journal of dental research, international endodontic journal | USA, China, Germany | University of Texas Health Science  Center, San Antonio, TX; University of North Carolina, Chapel Hill, NC; Peking University, Beijing, China | in vitro, reviews, case report  Type of study: In vitro, Reviews, Case report, Experimental animal, Quasi-experimental, Retrospective cohort, Case series, Prospective cohort, Single-arm longitudinal (before-after design) |
|  | WoS | Implant Dentistry | Peri-implant tissue healing and health, Implant success/failure, Biomechanics, Augmentation procedures and grafts, Implant loading, Surgical aspects, Esthetics, Orthodontic implants, Prosthodontic aspects, Preoperative imaging CBCT, Impact of patient’s general health | Lang NP, Buser D, Berglundh T | Clinical Oral Implants Research, International Journal of Oral & Maxillofacial Implants, Journal of Clinical Periodontology | USA, Sweden, Switzerland | University of Göteborg, University of Bern, Loma Linda University, University of Leuven, University of Texas Health Science Center at San Antonio | Study Design: Clinical (Case series, Poor quality cohort, Cohort, Cross sectional, RCT); Review (Narrative, Systematic); Basic (In vivo, In vitro); Proceedings papers |
|  | Scopus and WoS | Periodontology |  |  | Scopus: Journal of Periodontology, Journal of Clinical, Periodontology, Periodontology 2000  Wos: Journal of Periodontology, Journal of Clinical Periodontology, Periodontology 2000, Journal of Dental Researc |  |  | Article type in scopus: Article, review short paper  Article type in wos: Article, review, editorial, proceding paper, note |
|  | WoS | Oral, And Maxillofacial Trauma | Therapy, Prognosis, Epidemiology, Prevention, Diagnosis ,Pathogenesis,Combined  Dental trauma research has been a more active field than has research in oral and maxillofacial trauma. | Andreasen JO, Andreasen FM, Trope M | Dental Traumatology, journal Oral and Maxillofacial Surgery, International Journal of Oral Surgery | USA, Denmark, Sweden | University Hospital of Copenhagen, Denmark; Karolinska Institute, Sweden; University of Texas, USA | Study material (Patients, Animals, In vitro/Ex vivo, Not applicable)  Original research, review/meta-analysis, case report/case series |
|  | WoS | Cleft Lip And/Or Palate. | syndromes and Pierre Robin sequence, infant orthopedics, alveolar bone grafting, growth, tooth anomalies/ formation, and occlusion, speech and otorhinolaryngology, psychology and quality of life, epidemiology; anatomy, primary surgery, multicenter outcome studies, syndromes and Pierre Robin sequence, infant orthopedics | Semb G, Shaw WC, Asher Mc-Dade C, Plint DA | The Cleft Palate-Craniofacial Journal, American Journal of Orthodontics and Dentofacial Orthopedics, Journal of Oral and Maxillofacial Surgery | USA, UK, Denmark | University of Iowa, Iowa City, IA, USA; Montefiore Medical Center, Bronx, NY, USA; University of North Carolina, Chapel Hill, NC, USA |  |
|  | WoS | Dentistry | Periodontology, Implantology, Adhesive restorations, Bone morphology/histology, Endodontics, Caries, Oral medicine/pathology, Orthodontics, Saliva/Biochemistry, Pain dysfunction/orofacial pain syndrome, Oral hygiene, Pediatric dentistry, Behavior management | Socransky SS, Lindhe J, Löe H, Slots J, Haffajee AD | Journal of Clinical Periodontology, Journal of Periodontology, Journal of Dental Research |  |  | clinical, basic  evidence level: 5, 4, 3, 2, 1  Methodological design: Case series, Narrative review/expert opinion, Classifications or tools for evaluating results, Case–control studies, Animal, Cohort studies, In vitro, New material or technique, Randomized clinical trials, Systematic review/meta-analysis, Case report |
|  | WoS | Orthodontics | Implants, Airway resistance, Biomechanics and biology, Orthodontic appliance, functional, Class II correction, Temporomandibular joint/temporomandibular disorders, Demineralization, Root resorption, Palatal expansion, Bracket bonding and removal , Cephalometry, Growth and development, Assessing index, Stability and relapse of mandibular treatment, Cleft lip and/or palate, Auto-dental transplantation, Components of malocclusion class, Ectopically erupting maxillary canine, Vertical disorder, Cleft lip and/or palate, Auto-dental transplantation, Components of malocclusion class, Extraction and nonextraction orthodontic therapy, Social attractiveness, Soft-tissue facial profile, Mastication and malocclusion, Wire, Deep overbite correction, Injuries during orthodontic treatment, Orthodontic appliance, chin cup, Skeletal asymmetry, Skeletal Class III malocclusion, Two-phase treatment, Friction, Human molecular genetics approach to familial tooth agenesis | McNamara JA, Little RM, Bell WH | American Journal of Orthodontics and Dentofacial Orthopedics, The Angle Orthodontist, and European Journal of Orthodontics | USA, Sweden, Japan | The University of Michigan, United States, University of Washington, University of Oslo | Clinical experience, basic, review  level IV and level V evidence.  study design: Descriptive (Case series, Cross-sectional, Case report), Observational (Cohort, Case-control study), Expert opinion, Experimental (Randomized, controlled trial) |
|  | WoS | Endodontic | Endodontic microbiology, Leakage, MTA, Canal instrumentation, Irrigant, Physicochemical properties ofdentin pulp complex, Intracanal dressing, Restoration, Smear layer, Success and failure studies, Obturation, Traumatic dental injuries, Root canal anatomy, Pulp capping, Retrograde materials success/failure, Bleaching, Root resorption, Radiology | Torabinejad M, Ford TRP, Sundqvist G | Journal of Endodontics, journals Oral Surgery Oral Medicine Oral Pathology oral Radiology and Endodontology, International Endodontic Journal, Endodontics & Dental Traumatology. | USA, Sweden, Great Britain, Switzerland | Loma Linda University in California, University of Umea, University of Zurich, and University of North Carolina | science, clinical and nonsystematic reviews  Uncontrolled case series with level IV of evidence and narrative reviews with level V of evidence were the most frequent types of study design.  study design: Review, Case series, Case control, Nonrandomized controlled cohort, Cohort |
